# Oral health of 12-year-old children in Quito, Ecuador: a population-based epidemiological survey

**DOI:** 10.1186/s12903-019-0863-9

**Published:** 2019-08-14

**Authors:** Edgard Michel-Crosato, Daniela Prócida Raggio, Alba Narcisa de Jesus Coloma-Valverde, Edisson Fernando Lopez, Patricia Lourdes Alvarez-Velasco, Marco Vinicio Medina, Mariela Cumanda Balseca, Maritza Del Carmen Quezada-Conde, Fernanda Campos de Almeida Carrer, Giuseppe Alexandre Romito, Maria Ercilia Araujo, Maria Gabriela Haye Biazevic, Mariana Minatel Braga, Maristela Vilas Boas Fratucci, Fausto Medeiros Mendes, Antonio Carlos Frias, Claudio Mendes Pannuti

**Affiliations:** 1 Graduate Program in Dental Sciences, School of Dentistry, University of Sao Paulo, Av Lineu Prestes, 2227, São Paulo, 05508-000 Brazil; 2 School of Dentistry, Universidad Central del Ecuador, Quito, Ecuador

**Keywords:** Oral health, Dental caries, Dental trauma, Malocclusion, Gingival bleeding, fluorosis, epidemiological surveys

## Abstract

**Background:**

There is a paucity of population-based surveys on oral health conditions in Ecuador. Thus, the aim of this study was to conduct an epidemiological survey with a representative sample of children aged 12 years from public schools of Quito, Ecuador. The aim of this initial report was to describe the methodology used in the survey, as well to present results regarding calibration procedures and prevalence of oral-health related outcomes.

**Methods:**

We invited 33 public schools’ coordinators from the urban area of Quito, and 1100 children (12 years old) to take part in this study. Six trained and calibrated examiners conducted clinical examinations using oral mirrors and ball-ended probes to assess: dental caries, traumatic dental injuries, malocclusion, gingival bleeding, presence of calculus and fluorosis. Children also responded a questionnaire on Oral Health-Related Quality of Life (OHRQoL). Individual sociodemographic data was collected through a questionnaire sent to parents. Moreover, some contextual data on school environment (infrastructure conditions, promotion of health practices and negative episodes) were also evaluated. Prevalence values, crude and weighted by sampling weights, and 95% confidence intervals (95%CI) were calculated.

**Results:**

Nine hundred and ninety-eight children from 31 schools were examined from March to May 2017. The adjusted prevalence values (95%CI) for the six outcomes evaluated were: dental caries = 60.3% (55.3 to 65.0%); traumatic dental injuries = 20.7% (17.2 to 24.8%); dental fluorosis = 63.7% (58.5 to 68.5%); gingival bleeding = 92.0% (87.1 to 95.2%); presence of calculus = 69.9 (60.5 to 77.9%); and malocclusion = 25.8% (21.8 to 30.3%). Adjusted mean of number of decayed, missed or filled permanent teeth (DMF-T) was 1.61 (1.37 to 1.84). Results on OHRQoL and other contextual variables will be reported in other articles.

**Conclusion:**

The prevalence of the majority of oral health problems in 12-year-old children from public schools in Quito-Ecuador was compatible with those observed in other similar cities. However, periodontal health and fluorosis seem to be highly prevalent in children from Quito.

**Electronic supplementary material:**

The online version of this article (10.1186/s12903-019-0863-9) contains supplementary material, which is available to authorized users.

## Background

Epidemiological studies are essential to perform population diagnosis of some diseases. It is also important to investigate treatment needs of the population [1]. Moreover, cross-sectional studies can draw some lines for association between explanatory variables and the diseases occurrence in easier and cheaper ways when compared to longitudinal studies [2]. Although it is not possible to stablish causal relationship among the investigated factors and the disease, cross-sectional studies can be an important preliminary evaluation before planning cohort or case-control studies [2, 3]; hence this study design is considered as the initial step in science of unexplored areas [2].

For oral health, epidemiological surveys are important to identify populational status regarding some diseases and treatment needs, to monitor trends of diseases when the survey are periodically performed and draw the attention for policy makers and also for the population [3–6]. The World Health Organization (WHO) states that regular evaluations of oral health conditions have revealed important trends in oral health, especially in children [6]. Therefore, surveys in representative samples are the first step towards better oral health.

Despite this recognized importance, some large cities do not have representative well-designed population-based epidemiological surveys of oral health conditions. One example is Quito, capital of Ecuador. Although Quito is an important city in Latin America, only anecdotal studies have been published in journals with international circulation. Some examples were the Atahualpa project, where the authors investigated the association of edentulism and systemic disorders in persons aged 60 years or older, in a countryside city of Ecuador. The outcomes were poor cardiovascular health [7] and worse cognitive performance [8].

In another study conducted in Ecuador, the authors compared caries prevalence and severity in indigenous and non-indigenous children living in Francisco de Orellana e Aguarico in the northeast region (Amazon) of the country, in 6- to 12-year-old children [9]. They found high caries experience and prevalence in both populations, and no difference among indigenous or non-indigenous [9]. Although this is the only paper published in an international journal about caries in Ecuador, the sample is very specific and impairs generalization regarding dental caries in other Ecuadorian regions. Recently, another cross-sectional research was conducted to evaluate the prevalence, severity and periodontal attachment loss in 144 15- to 19-year old 144 adolescents from six Latin-American cities, including Quito. The authors observed that adolescents living in Quito presented higher prevalence value of at least one site with clinical attachment loss than adolescents from other cities [10].

Therefore, there is a lack of epidemiological data for oral health conditions in Ecuador, mainly in children. Due to this scenario, the aim of this study was to perform an epidemiological survey on oral health conditions in a representative sample of children aged 12 years old studying in public schools of urban area of Quito, capital of Ecuador. The objective of this first manuscript was to detail the methodology used in the survey, as well as to present the results regarding examiners’ calibration and prevalence obtained for the oral health conditions evaluated.

## Methods

### Study design and ethical aspects

This study is part of an inter-institutional PhD program supported by Coordination of Improvement of Higher Education Personnel (CAPES), a Brazilian government agency responsible for evaluating and funding graduate programs. The research involves supervisors from School of Dentistry, University of Sao Paulo (FOUSP), and the PhD students, who are also working as senior lecturers of Central University of Ecuador (UCE).

**Fig. 1:**
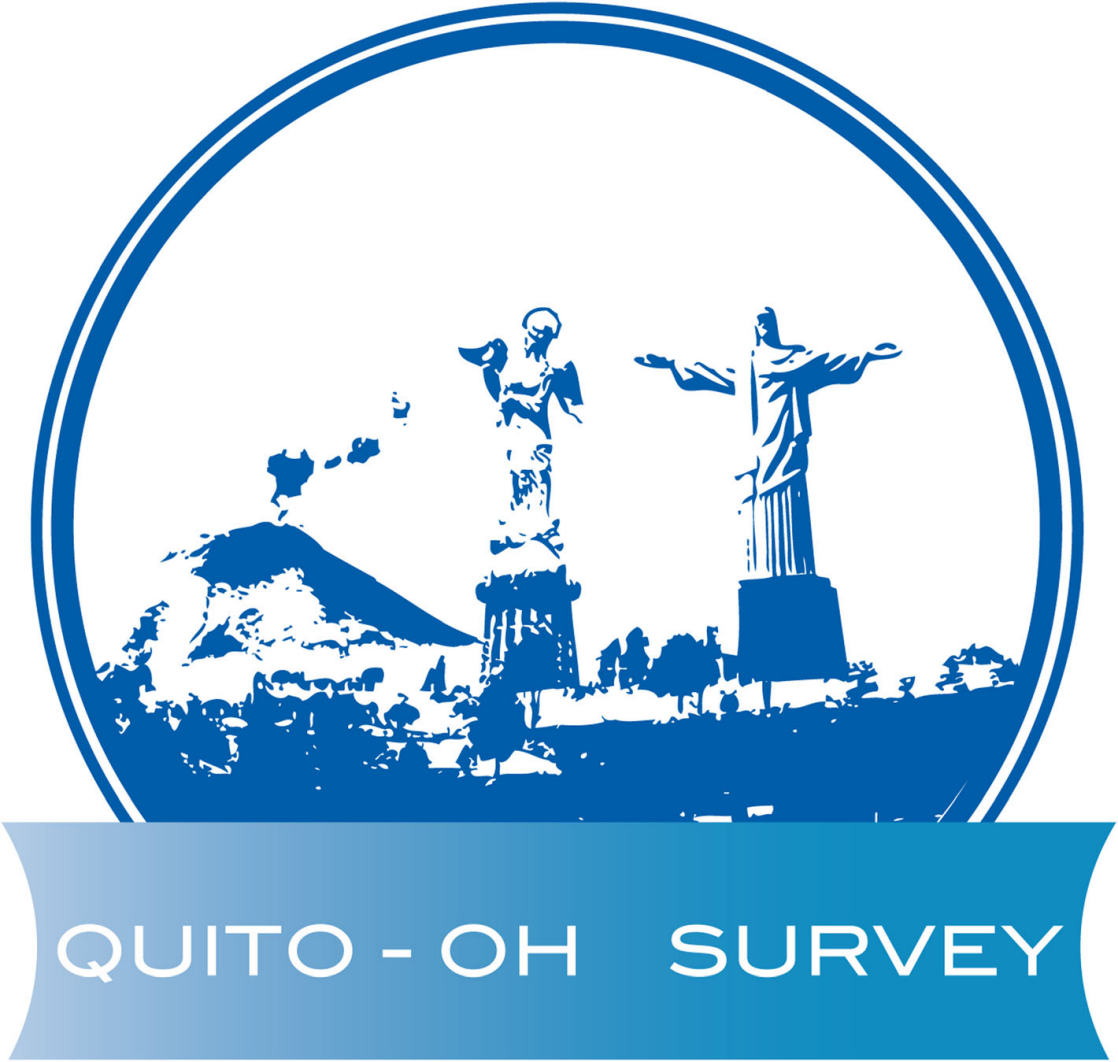
QUITO-OH survey logotype

This descriptive cross-sectional study is a population-based epidemiological survey with a representative sample of 12-year old children studying in public schools from the urban area of Quito, Ecuador. This large project was named “QUITO Oral Health Survey” (QUITO-OH Survey). A collaborative group (QUITO-OH Survey collaborative group) with all persons who helped in the different phases of the study was created, and the QUITO-OH survey logotype is presented in Fig. 1.

The protocol was approved by the Research Ethics Committee from both schools (FOUSP, CAAE # 61903416.8.0000.0075; and UCE, # 399-CE-UCE-2016) and it is in accordance with the international Helsinki’s Declaration. Legal guardians of children participants of the study were contacted and signed an inform consent. Further, children signed an assent form prior to their inclusion. This manuscript was written following the recommendations of the guideline ‘Strengthening the Reporting of Observational studies in Epidemiology (STROBE)’. The STROBE checklist can be found as Additional file 1.

### Setting, sampling and participants

The study was conducted in public schools of the urban area of Quito, Ecuador. The country has approximately 17,000,000 inhabitants (projection for 2019), and around 30% of this population is under 15 years-old. The Human Development Index is 0.752 (ranked 86th in the world) and the gross national income per capita of 11,350.00 PPP dollars.

Quito is the capital and second most populous city in Ecuador. The city has an area of 372.4 km^2^ and the altitude of 2850 m. It has about 2,6 million inhabitants (projection for 2019), and a population density of 7200 people living per km^2^_._ The city has 156 public schools in the urban area, with approximately 134,000 students and about 17,000 aged 12 years.

A two-stage cluster sampling method was used. First, we randomly selected 33 schools, stratified by area and proportion of 12-year-old students, using a simple lottery system. The selection was carried out to obtain similar number of schools of each area of Quito (Southern, Central and Northern areas), since number of schools in each region is similar. Therefore, we randomly selected 11 schools in each area. As the WHO [6] states that in cities with more than 50,000 inhabitants is necessary at least 20 places to collect the data, this number of schools was adequate for our survey. After this first stage, we randomly selected the students from these schools. Those procedures were followed to prevent selection bias and to guarantee the representativeness of the sample.

We planned to evaluate several outcome variables related to oral health problems. Thus, we adopted an estimated prevalence value of 50% for the calculation of the minimal sample necessary to the survey. Considering a population of 17,000 children aged 12 years, a standard error of 4%, a 95% confidence interval (95%CI) and a correction factor of 1.6 to consider the design effect for complex sample surveys, we reached a minimum of 928 children. To deal with non-responses and refusals to participate we added up around 20% to this number. Therefore, 1100 children were randomly selected from schools whose coordinators accepted to participate of the survey.

The eligibility criteria were: (i) children studying in a public school of the urban area of Quito, and (ii) children who were born between April 2004 and March 2005. This range was determined because the examinations started in March 2017. To be included in the study, children’s legal guardians should sign the inform consent, and children should sign the assent form.

### Training and calibration of the examiners

Six examiners, all senior lecturers at the UCE (ANJC-V, EFL, PLA-V, MVM, MCB and MDCQ-C), conducted all dental examinations. Moreover, 12 undergraduate students were invited to help in the study. Their roles were to register the data from clinical examinations, to interview the children regarding the Oral Health-Related Quality of Life (OHRQoL) questionnaire or to organize the order of examinations. Two months before the start, examiners and the entire staff took part of training and calibration sessions. Two benchmark examiners (ACF and MVBF) were responsible for this procedure. The calibration sessions lasted 10 days.

Previously to training and calibration sessions, the examiners read the book published by WHO about the basic methods for oral health surveys [6], as well as some e-learning support materials. During the sessions, examiners and assistants attended theoretical lectures gave by the benchmark examiners (a total of 40 h). The lecture content was based on organization, methods and indexes used during the survey. They also observed clinical examples of each oral health condition.

After this period, clinical evaluations were conducted. For calibration sessions, children (12 to 14 years old) attending in a school (not included in the main study) participated. The “consensus technique” was used for the calibration [11, 12]. For this technique, two or three examiners evaluated the same child, and the results were compared and discussed among them and the benchmark examiners. After the discussion, they reached a consensus, and this result was considered the “reference standard”. Then, the other examiners performed the clinical examinations, and their results were compared with the reference standard reached by the prior consensus [12]. The calibration sessions before the main study only finished when all inter-examiner reproducibility reached a kappa value higher than 0.700 for all oral health conditions. The note-takers and other assistants also attended these sessions.

### Data collection and variables

For the data collection during the epidemiological survey, the examiners and assistants were divided into 3 teams with 2 examiners, 2 note-takers, 1 interviewer and 1 person to help the participants in all stages. Each team was responsible to perform the examinations in schools located at each area of Quito: Southern, Central and Northern. The staff members went to the schools and returned to each school up to 3 times to recover students who were selected but were absent in the days of the examination, or whose parents have not signed the consent form. After 3 unsuccessful attempts in examining these missed children, they were considered as drop-outs.

Children were examined in isolated rooms with artificial illumination. They used plane buccal mirrors and ball-ended probe. When necessary, examiners dried the teeth with gauze pads. The same examiner evaluated all oral health conditions in each child.

The variables related to the children’s oral health evaluated in the present study were dental caries, traumatic dental injuries, malocclusion, gingival bleeding, presence of calculus and fluorosis. For dental caries evaluation, we used the Decayed, Missed, and Filled permanent teeth (DMF-T) criteria, as proposed by WHO [6]. All teeth were evaluated, and each tooth was recorded as sound, decayed (cavitated caries lesion), missed due to caries or filled. Then the DMF-T value was attributed for each child; furthermore, children were classified as with no caries (DMF-T = 0) and with dental caries (DMF-T > 0).

For assessing traumatic dental injuries the O’Brien criteria [13] was used (as recommended by WHO) [6]. Only upper and lower incisors were evaluated, and they were classified in: (0) no traumatic dental injury; (1) fracture restricted to the enamel; (2) fracture reaching the dentin; (3) fracture and sign of pulpal involvement; (4) with no fracture, but with any sign of pulpal involvement; (5) tooth missed due to traumatic dental injury; (6) other types of traumatic dental injuries. Children were considered as having traumatic dental injuries when at least one tooth presented an enamel fracture or a more severe condition.

The outcomes related to the periodontal health were evaluated using the Community Periodontal Index (*CPI*). The examiners evaluated six sites in index teeth, one in each sextant [6]. Teeth evaluated were all first permanent molars (16, 26, 36 and 46), and one upper and one lower central incisor (11 and 31, respectively). The examiners evaluated sites for presence of gingival bleeding (no or yes), and calculus (no or yes). Presence of periodontal pocket was not assessed as this condition is not common in this age. The presence of gingival bleeding and calculus was defined separately, when 1 sextant or more presented the condition.

The malocclusion was evaluated using the Dental Aesthetics Index (DAI) [14]. DAI is a quantitative index; the examiner evaluates 10 occlusal characteristics and give scores for each one according different weights. Then, the scores were categorized according predetermined cut-offs in: (i) normal occlusion; (ii) definite malocclusion; (ii) severe malocclusion; or (iv) handicapping malocclusion. In our study, children were considered as with malocclusion when they were classified with severe or handicapping malocclusion [14].

Finally, examiners evaluated the children for dental fluorosis using the Dean index [6]. Examiners coded the children based on two most severe affected teeth. Children were classified as with no fluorosis (normal or questionable teeth) or with fluorosis (very mild, mild, moderate or severe fluorosis) [6]. Parents of children who presented severe oral health problems were orientated to look for dental assistance in the dental school or in a public health center.

Other variables were collected. Socioeconomic and demographic data were collected using a structured questionnaire answered by the parents. This questionnaire contained information on family income (total income converted in Ecuadorian Minimum Wage), mother’s and father’s level of education (number of years of regular study), number of residents in the house and number of rooms in the house.

Regarding the OHRQoL children answered the Spanish version [15] of the short form of Child Perceptions Questionnaire for 11–14-year-olds (CPQ11–14) [16]. We used the version translated and validated for Spanish language by Mexican researchers [15], doing few adaptations for Ecuadorian context. The short form comprises 16 questions permitting answers ranging from 0 to 4. These 16 questions are distributed in four domains (4 questions each): oral symptoms, functional limitations, emotional and social well-being. Therefore, the total scores can vary from 0 to 64, with higher scores indicating a more negative impact on OHRQoL. Data on scholar performance of the participants were also recorded, considering grades in Mathematics and Language (Spanish), and well as the number of days missed. These data were gathered considering the last regular annual period.

Moreover, some characteristics of schools that participated in the study were evaluated. The school coordinators provided this information. Contextual data collected was related to physical conditions of the school (patio area, type of patio floor, number of students per class, and presence of stairs and ramps), promotion of health practices (offering of healthy meals, permitting the students brushing their teeth, and allowing sports practice after regular class time), and negative episodes that occurred in the school (vandalism, theft episodes or violence between the students).

### Data analysis

Besides the organization of the epidemiological survey, the present study focused on the calibration of the examiners and on the prevalence of oral health problems in 12-year scholars from Quito. Results related to other variables (OHRQoL, school performance and contextual variables) will be presented and analyzed in further manuscripts.

The inter-examiner agreement obtained by the examiners in the calibration sessions was calculated for all outcomes at the children level (having or not having the condition). Therefore, we considered dichotomous results and used Cohen’s kappa test to calculated the reproducibility values with the respective 95%CIs. Moreover, we derived the percentage agreement among the examiners.

In the main study, crude prevalence values of oral health problems were calculated. However, we also calculated the prevalence (95%CIs) adjusting the values for the sampling weights. For this, weights for each school were calculated considering the non-response rates, considering the eligible and actual number of children examined. This procedure was similar to the method used in the Brazilian Oral Health Survey, named SBBrasil. More details can be found elsewhere [17, 18]. Specifically, for dental caries, mean and standard deviation of DMF-T values were calculated, also adjusting for the sampling weights. A statistical package (Stata 13.0, StataCorp LP, College Station, USA) was used for the analysis.

## Results

In the calibration sessions, 64 children from a non-included school were examined to calculate the inter-examiner reproducibility. The number of examinations varied according to the oral health problem; this inconsistency was due to the consensus method used in this part of the study. Number of examinations for each oral health condition, percentage agreement and kappa values are presented in the Table 1.

**Table 1:** Results obtained regarding the inter-examiner reproducibility among the 6 examiners obtained during the calibration sessions

Oral health condition	Number of Examinations	Percentage agreement ^a^	Kappa value ^a^	95% CI
Dental caries	376	97.1	0.94	0.92 to 0.95
Gingival bleeding	234	92.2	0.73	0.65 to 0.82
Presence of calculus	234	92.5	0.85	0.79 to 0.89
Malocclusion	256	93.6	0.87	0.75 to 0.97
Dental fluorosis	272	91.2	0.83	0.69 to 0.85
Traumatic dental injuries	200	98.8	0.89	0.79 to 0.98

^a^agreement with the consensus obtained with other examiners

*95%CI* 95% confidence intervals

**Fig. 2:**
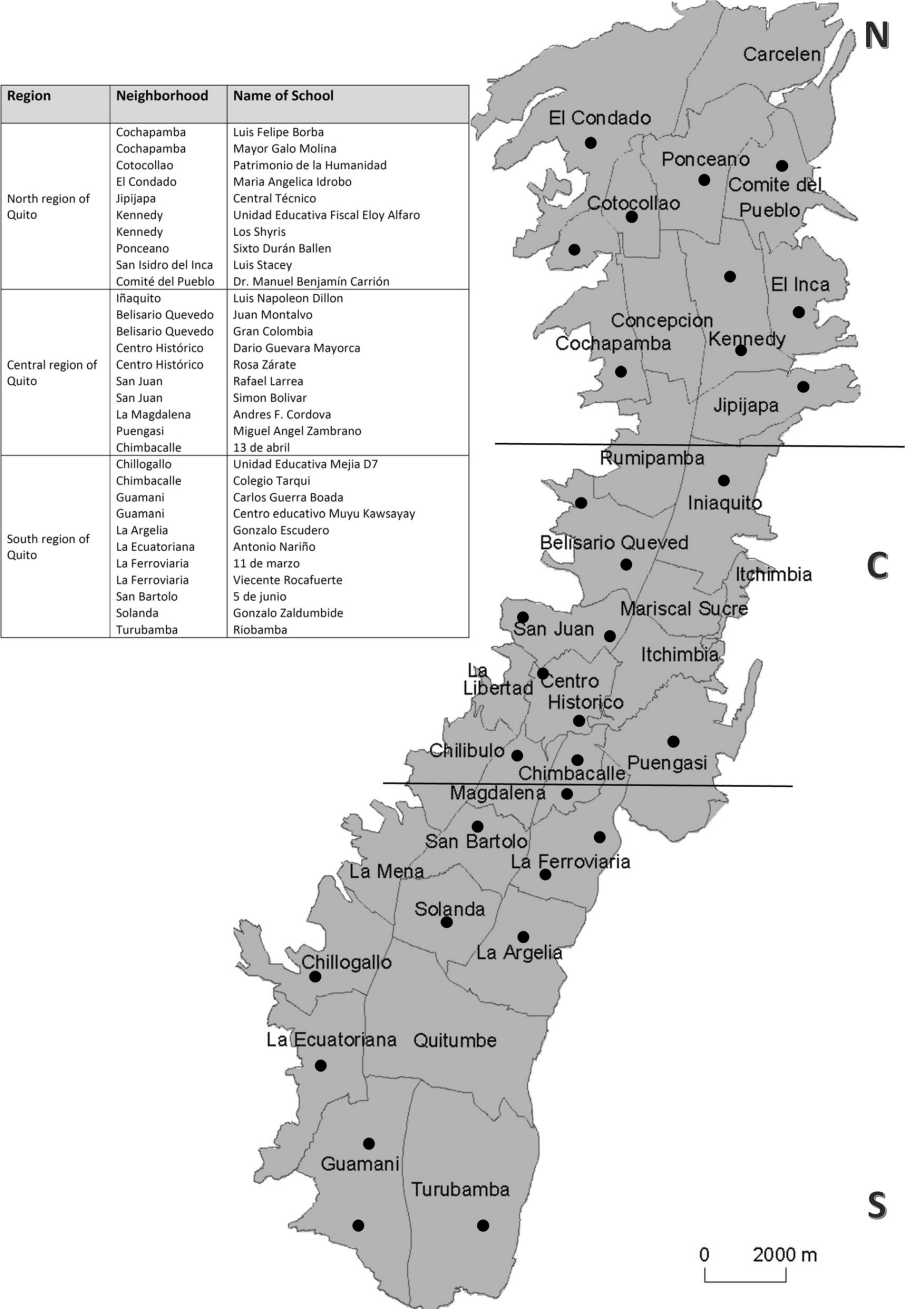
Map of Quito indicating the areas of schools participating (source: adapted from Wikimedia Commons. Author: H. F. Lopez (2000). Source: http://commons.wikimedia.org/wiki/File:Mapa_de_Parroquias_de_Quito.jpg

For the main study, coordinators of 31 school (from 33 invited, 93.9%), accepted to participate in the study. The location of the schools was well-distributed in Quito areas: 10 in the Northern and Central area and 11 in the Southern area (Fig. 2). Then, 12 year-old children from these schools were randomly selected. From 1100 children selected and invited, 998 children participated of the study (positive response rate of 90.7%). The reason for drop-outs were: not returning the consent form (96 children), absence in 3 examinations dates (4 children) or children’s refusal to be examined (2 children).

From 998 participants, 350 students were from schools located at the North region, 371 were from central and 277 students were from schools located at the south region of Quito. The descriptive analysis regarding sociodemographic characteristics of the sample are described in Table 2. Most participants were girls (55.5%), and more than 50% of children’s families were classified with better conditions for all socioeconomic variables (Table 2).

**Table 2:** Demographic and socioeconomic characteristics of children who participated in the epidemiological survey

Variables	N ^a^	%
Sex		
Female	554	55.5
Male	444	44.5
Family income		
Up to 1 EMW ^b^	434	45.2
More than 1 EMW	526	54.8
Mother’s level of education		
Up to 8 years of formal education	343	34.4
More than 8 years of formal education	654	65.6
Father’s level of education		
Up to 8 years of formal education	343	34.5
More than 8 years of formal education	650	65.5
Household crowding		
Up to 1.7 persons per room	603	60.8
More than 1.7 persons per room	389	39.2
Total	998	100

^a^Some variables do not total 998 children due to missing data

^b^*EMW* Ecuadorian minimum wage (about USD 375.00/ month during the period of data gathering)

Considering the data related to oral health problems, the less prevalent condition was traumatic dental injuries (adjusted prevalence of 20.73%) and the most prevalent oral health problem was gingival bleeding, with an adjusted prevalence of 93.2% (Table 3). Severe or handicapping malocclusion occurred in about 25% of Ecuadorian children, and about 60% of participants presented dental caries and/or fluorosis. Mean of DMF-T was approximately 1.6 (Table 3).

**Table 3:** Prevalence of oral health problems evaluated in the epidemiological survey in 12-year-old children from Quito, Ecuador, and caries severity presented as mean of Decayed, Missed or Filled Teeth (DMF-T)

Oral Health problems	Unadjusted prevalence (%)	95% CI	Adjusted prevalence ^a^ (%)	95% CI
Dental caries	58.5	55.4 to 61.5	60.3	55.3 to 65.0
Dental fluorosis	63.5	60.5 to 66.4	63.7	58.5 to 68.5
Traumatic dental injury	18.3	16.1 to 20.9	20.7	17.2 to 24.8
Gingival bleeding	93.2	91.4 to 94.6	92.0	87.1 to 95.2
Presence of calculus	73.5	70.7 to 76.2	69.9	60.5 to 77.9
Malocclusion	25.6	22.3 to 28.4	25.8	21.8 to 30.3
Caries severity	Unadjusted Mean (SE)	95% CI	Adjusted Mean * (SE)	95% CI
DMF-T	1.52 (0.06)	1.41 to 1.64	1.61 (0.11)	1.37 to 1.84

^a^Values adjusted by the sampling weights 95%

*95%CI * confidence intervals, *SE* Standard error

## Discussion

Identification and monitoring of oral health status of a population is important to identify treatment needs and to evaluate periodically the impact of public oral health programs in reducing these problems [3, 5, 17, 19, 20]. This can be demonstrated by the large number of studies reporting the prevalence of oral health problems [4, 17, 18, 21–24]. Despite this recognized significance some countries have only scarce data on prevalence of oral health conditions. An example is Ecuador, an important South American country. According the WHO oral health databases [24], last information on dental caries in the country was obtained in the last century, and this information was obtained from a study in a non-representative sample [9]. There was a paucity of prevalence data on other oral health problems in Ecuador, mainly in children.

Due to this scenario, the present study was performed to collect data on prevalence of several oral health conditions in scholars of Quito, capital city of Ecuador. This first report intends to describe methodological aspects of this survey, including planning, organization, calibration and data collection. We also presented the inter-examiner agreement values and prevalence figures of the oral health conditions evaluated. Additional manuscripts will be prepared detailing each oral health outcome, describing full data on prevalence and severity of the conditions, and analyzing the association with individual and contextual variables. Moreover, impact of these oral health problems on children’s OHRQoL and school performance will be also further analyzed.

The methodological issues planned in the present study were completed successfully. The calibration sessions lasted 10 days, and inter-examiner kappa values higher than 0.70 was obtained for all outcomes. From 33 schools invited, coordinators from 31 (93.9%) schools agreed to participate of the study; moreover, a positive response rate of 90.7% was obtained with the children invited to participate in the study. Therefore, we can consider that the data obtained is representative of scholars of Quito studying in public schools of urban area of Quito.

Regarding the prevalence values observed in this population, around 60% of children had dental caries. This figure is compatible with the overall caries prevalence observed in a systematic review recently published. In this review, the authors compiled studies conducted in Latin America after 2000 [20]. Considering our results, we could observe that dental caries in Quito follow the same pattern for similar cities, at least in 12-year-old children. Moreover, we could speculate that this similarity gives support to our statement on representativeness of our study.

Considering the DMF-T, unfortunately the systematic review has not presented data on this parameter [20]. We observed a DMF-T mean of 1.6. Considering the Brazilian results obtained in 2010, this value was lower, since in Brazil was observed a DMF-T mean of 2.04 [25]. Nevertheless, it was similar to the DMF-T value obtained in big cities, for instance São Paulo (DMF-T around 1.4), the most populous Brazilian city [26].

Furthermore, we could observe similarity in the prevalence of traumatic dental injuries between our study and data obtained in other Latin-American countries. Another comprehensive systematic review on prevalence data of traumatic dental injuries in countries from Latin America and Caribbean found an overall prevalence of 18.6% [21], while we observed that about 20% of children in our study had at least an enamel fracture.

Regarding the malocclusion, we used the DAI method, that is the index proposed by the WHO [6] to be used for epidemiological purposes. Comparing results obtained in Quito with findings obtained in Latin American cities, we observed a prevalence of severe or handicapping malocclusion of around 25%, which is similar to studies performed in some Brazilian cities [27–29] or the value observed in the Brazilian Oral Health Survey, conducted in 2010 [30]. Comparing with other regions from Latin America, prevalence value observed in Quito was slightly higher than those observed in Uruguay or Mexico [31, 32]. However, these latter studies were conducted with older persons, what can explain the differences.

Therefore, considering dental caries, traumatic dental injuries and malocclusion, figures observed in Quito were compatible with prevalence values observed in other regions from Latin America. On the other hand, considering other oral health outcomes evaluated (fluorosis and periodontal health), Quito presented a high prevalence. For example, considering fluorosis, more than one half of scholars from Quito presented some degree of fluorosis. This is higher than the prevalence of fluorosis observed in Brazil (less than 20%) [33, 34], except for some regions with endemic fluorosis due to high concentration occurring in the natural groundwater [35, 36]. Furthermore, the prevalence observed was higher than the pooled prevalence values observed in a systematic review when the fluoride concentration of water supply was lower than 0.7 mg/L (around 17%) or until 1.2 mg/L (around 27%) [23].

Nevertheless, results from Quito were similar to regions where the water supply presented higher concentrations of fluoride (higher than 1.3 mg/L) [23]. Moreover, other reports from other Latin American countries, for instance in Colombia [37, 38] and Mexico [39, 40]. Quito and these other places are in high altitude, and all of them present fluoride added in the salt. However, the reasons for high prevalence of fluorosis is unclear and it should be explored more deeply in further studies.

About periodontal parameters, almost all children examined had at least one sextant with gingival bleeding after probing. Our results differ from other epidemiological surveys. For instance, in the Brazilian Oral Health Survey, prevalence of gingival bleeding was about 37% for 12 year-old children, and almost 50% for adolescents from 15 to 19 years old [41]. The same trends were observed considering presence of calculus; the prevalence observed in adolescents were 36.1% in Brazil [41], and in our study, we observed a prevalence of around 70%. On the other hand, studies performed in some Brazilian cities observed prevalence of gingival bleeding similar to that observed in Quito [42–44]. These discrepancies could be due to methodological aspects, since the Brazilian survey involves higher number of examiners. The necessity of high numbers of examiners involves higher difficult in guaranteeing a good accuracy of examinations during entire study. Other unknown reasons, such as, population characteristics, could also explain the differences observed. In fact, in an extensive survey conducted in six Latin American cities to evaluate occurrence of clinical attachment loss in 15 to 19 year-old adolescents, Quito presented the worst conditions considering all cities [10]. However, the actual reasons to explain these high prevalence values of periodontal parameters in Quito should be investigated in further studies.

The next steps regarding this project is to analyze and report individually the prevalence and severity data of each oral health problem evaluated, as well as, to evaluate individual and contextual variables associated with these outcome variables. Moreover, influence of these oral health problems (and other individual and contextual variables) on children’s OHRQoL, measured using the CPQ11–14, will be analyzed, as well as the influence of these variables on children’s school performance, measured through grades obtained in Mathematics and language, and days missed in the last year. Other objective is to extend this survey in a national level. The experience achieved with the participation in this study will permit that the UCE lecturers organize, train and calibrate examiners from other Ecuadorian cities.

## Conclusions

In conclusion, considering the main objective of this project, that was to conduct an epidemiological survey with a representative sample of 12-year-old children from the most important city of Ecuador, the goal was accomplished. The study was organized with six examiners who reached good agreement values and finished all examinations in a period of 3 months. Moreover, the sample is well-distributed in all areas of Quito, and the positive response rate was higher than 90%. The prevalence figures observed for most oral health problems were similar to those reported in other similar Latin American cities. The discrepancies were observed for fluorosis and periodontal health; both outcomes presented higher prevalence values compared with those observed in other places.

## Additional files

Additional file 1: STROBE checklist (PDF 140 kb)

## Data Availability

The datasets used and/or analyzed during the current study will be available on the Public Repository of the University of São Paulo, when all manuscripts related to this study are accepted for publication.

## Abbreviations

95%CI: 95% confidence interval
CAPES: Coordination of improvement of higher education personnel
CPI: Community periodontal index
CPQ11–14: Child perceptions questionnaire
DAI: Dental aesthetics index
DMF-T: Decayed, missed, and filled permanent teeth
FOUSP: School of Dentistry, University of Sao Paulo
OHRQoL: Oral health-related quality of life
QUITO-OH Survey: Quito oral health survey
STROBE: Strengthening the reporting of observational studies in epidemiology
UCE: Central University of Ecuador
WHO: World Health Organization
