# Supplementary material for: Oral health of 12-year-old children in Quito, Ecuador: a population-based epidemiological survey
Source: BMC Oral Health. 2019 Aug 14;19:184. doi: 10.1186/s12903-019-0863-9 (PMC6694588; doi:10.1186/s12903-019-0863-9)
Supplement: Supplementary file 1 — STROBE checklist (PDF 140 kb) [file 12903_2019_863_MOESM1_ESM.pdf]

STROBE Statement—Checklist of items that should be included in reports of ***cross-sectional studies***

	Item No	Recommendation	Page No.
**Title and abstract**	1	(*a*) Indicate the study's design with a commonly used term in the title or the abstract	Page 1 and 4
		(*b*) Provide in the abstract an informative and balanced summary of what was done and what was found	Page 4
**Introduction**			
Background/rationale	2	Explain the scientific background and rationale for the investigation being reported	Page 5 and 6
Objectives	3	State specific objectives, including any prespecified hypotheses	Page 6, 2nd paragraph
**Methods**			
Study design	4	Present key elements of study design early in the paper	Page 6, 4th paragraph
Setting	5	Describe the setting, locations, and relevant dates, including periods of recruitment, exposure, follow-up, and data collection	Page 7, 1st paragraph
Participants	6	(*a*) Give the eligibility criteria, and the sources and methods of selection of participants	Page 7, 3rd, 4th and 5th paragraphs
Variables	7	Clearly define all outcomes, exposures, predictors, potential confounders, and effect modifiers. Give diagnostic criteria, if applicable	Page 9 to 11 section 'Data collection and variables'
Data sources/ measurement	8*	For each variable of interest, give sources of data and details of methods of assessment (measurement). Describe comparability of assessment methods if there is more than one group	Page 8 ('Training and calibration of the examiners') and Page 9 to 10 ('Data collection and variables')
Bias	9	Describe any efforts to address potential sources of bias	Page 7, 3rd paragraph
Study size	10	Explain how the study size was arrived at	Page 7, 3rd and 4th paragraph
Quantitative variables	11	Explain how quantitative variables were handled in the analyses. If applicable, describe which groupings were chosen and why	Page 9 to 10
Statistical methods	12	(*a*) Describe all statistical methods, including those used to control for confounding	Page 11 (section ' Data analysis')
		(*b*) Describe any methods used to examine subgroups and interactions	N/A
		(*c*) Explain how missing data were addressed	N/A

		(*d*) If applicable, describe analytical methods taking account of sampling strategy	Page 11, 3rd paragraph
		(*e*) Describe any sensitivity analyses	N/A
**Results**			
Participants	13*	(a) Report numbers of individuals at each stage of study—eg numbers potentially eligible, examined for eligibility, confirmed eligible, included in the study, completing follow-up, and analysed	Page 12, 1st paragraph
		(b) Give reasons for non-participation at each stage	Page 12, 1st paragraph
		(c) Consider use of a flow diagram	N/A. This information is described in the text (page 12, 1st paragraph)
Descriptive data	14*	(a) Give characteristics of study participants (eg demographic, clinical, social) and information on exposures and potential confounders	Table 2
		(b) Indicate number of participants with missing data for each variable of interest	Table 2 (footnote)
Outcome data	15*	Report numbers of outcome events or summary measures	Table 3
Main results	16	(*a*) Give unadjusted estimates and, if applicable, confounder-adjusted estimates and their precision (eg, 95% confidence interval). Make clear which confounders were adjusted for and why they were included	Table 3
		(*b*) Report category boundaries when continuous variables were categorized	N/A
		(*c*) If relevant, consider translating estimates of relative risk into absolute risk for a meaningful time period	N/A
Other analyses	17	Report other analyses done—eg analyses of subgroups and interactions, and sensitivity analyses	N/A
**Discussion**			
Key results	18	Summarise key results with reference to study objectives	Pages 13 to 15
Limitations	19	Discuss limitations of the study, taking into account sources of potential bias or imprecision. Discuss both direction and magnitude of any potential bias	Page 15, 1st paragraph
Interpretation	20	Give a cautious overall interpretation of results considering objectives, limitations, multiplicity of analyses, results from similar studies, and other relevant evidence	Pages 13 to 15
Generalisability	21	Discuss the generalisability (external validity) of the study results	Pages 13 to 15
**Other information**			
Funding	22	Give the source of funding and the role of the funders for the present study and, if applicable, for the original study on which the present article is based	Page 16, last paragraph

*Give information separately for exposed and unexposed groups.

Note: An Explanation and Elaboration article discusses each checklist item and gives methodological background and published examples of transparent reporting. The STROBE checklist is best used in conjunction with this article (freely available on the Web sites of PLoS Medicine at http://www.plosmedicine.org/, Annals of Internal Medicine at http://www.annals.org/, and Epidemiology at http://www.epidem.com/). Information on the STROBE Initiative is available at www.strobe-statement.org.
